# Pathogenicity of SARS-CoV-2 Omicron Subvariants JN.1, KP.2, and EG.5.1 in K18-hACE2 Transgenic Mice

**DOI:** 10.3390/v17091177

**Published:** 2025-08-28

**Authors:** Lila D. Patterson, Amany Elsharkawy, Hamid Reza Jahantigh, Zainab Nabi, Shannon Stone, Mukesh Kumar

**Affiliations:** 1 Department of Biology, College of Arts and Sciences, Georgia State University, Atlanta, GA 30303, USA; lpatterson37@gsu.edu (L.D.P.); aelsharkawy2@gsu.edu (A.E.); hjahantigh@gsu.edu (H.R.J.); znabi1@gsu.edu (Z.N.); sstone12@gsu.edu (S.S.); 2 Molecular Basis of Disease, College of Arts & Sciences, Georgia State University, Atlanta, GA 30303, USA

**Keywords:** COVID-19, SARS-CoV-2, variants of concern, omicron, EG.5.1, JN.1, KP.2, ACE-2 expressing mice, neuroinvasion, pathogenicity

## Abstract

The emergence of the SARS-CoV-2 JN.1 lineage in late 2023 marked a major shift in viral evolution. By January 2024, it had displaced XBB variants to become the dominant strain worldwide. JN.1 and its descendants are antigenically distinct from earlier Omicron subvariants, with approximately 30 additional spike mutations compared to XBB-derived viruses. The combination of these features alongside growing evidence of considerable immune evasion prompted the FDA to recommend that vaccine formulations be updated to target JN.1 rather than XBB.1.5. The continued dominance of JN.1-derived variants necessitates the characterization of viral infection in established animal models to inform vaccine efficacy and elucidate host–pathogen interactions driving disease outcomes. In this study, transgenic mice expressing human ACE2 were infected with SARS-CoV-2 subvariants JN.1, KP.2, and EG.5.1 to compare the pathogenicity of JN.1-lineage and XBB-lineage SARS-CoV-2 viruses. Infection with JN.1 and KP.2 resulted in attenuated disease, with animals exhibiting minimal clinical symptoms and no significant weight loss. In contrast, EG.5.1-infected mice exhibited rapid progression to severe clinical disease, substantial weight loss, and 100% mortality within 7 days of infection. All variants replicated effectively within the upper and lower respiratory tracts and caused significant lung pathology. Notably, EG.5.1 resulted in neuroinvasive infection with a significantly high viral burden in the brain. Additionally, EG.5.1 infection resulted in a significant increase in CD8^+^ T cell and CD11b^+^ CD11c^+^ dendritic cell populations in infected lungs.

## 1. Introduction

First identified in August 2023, the Omicron subvariant BA.2.86 is characterized by approximately 30 mutations within the spike protein compared to then-dominant XBB lineage viruses. Owing to its high degree of genetic divergence, BA.2.86 was quickly designated a variant under monitoring due to concerns of increased transmissibility and immune evasion; however it ultimately failed to gain significant traction within the population, displaying limited evasion from antibodies elicited by vaccination or prior infection [1,2]. The JN.1 variant evolved soon after, distinguished from its predecessor BA.2.86 by a single additional spike protein mutation, L455S [3,4]. Unlike BA.2.86, JN.1 spread rapidly. According to the U.S. Centers for Disease Control and Prevention (CDC), JN.1 accounted for less than 0.1% of sequences by the end of October 2023 [5]. However, by late December, its prevalence soared to 44% of SARS-CoV-2 sequences in the United States. JN.1 continued to outcompete existing XBB lineage variants, such as EG.5.1, becoming the dominant variant worldwide by the end of January. By April, nearly all the publicly available SARS-CoV-2 genetic sequences were derived from JN.1 [5,6].

The rapid displacement of XBB lineage variants suggests JN.1 possesses a significant growth advantage, likely driven by its highly mutated spike protein [7]. JN.1 is antigenically distinct from XBB.1.5, the primary target of updated vaccines at the time, demonstrating markedly increased evasion of neutralizing antibodies compared to previous Omicron variants. Contributing to the enhanced immune escape of JN.1, the characteristic L455S mutation is located at the epitope of the receptor-binding domain of Class I antibodies, conferring enhanced humoral immune resistance. Having retained the antigenic diversity of BA.2.86, JN.1 is similarly resistant to class II and III antibodies [1,3]. Interestingly, though its predecessor BA.2.86 displayed a remarkably high ACE2 binding affinity, there is a marked reduction in the ACE2 binding affinity for the JN.1 receptor binding domain. The substantial increase in resistance of JN.1 to neutralizing antibodies in part explains why JN.1 quickly became the dominant variant globally despite BA.2.86 failing to do so [3]. JN.1-derived variants, including KP.2, KP.3, and XEC, have independently evolved with mutations at F456L and/or R346T. Substitutions at these positions have been identified in previous SARS-CoV-2 variants, including XBB, BQ.1, and EG.5.1, and are associated with enhanced immune evasion owing to their location within target epitopes of neutralizing antibodies [3,7]. Studies demonstrate that sera from animals and humans having received the XBB.1.5 monovalent vaccine effectively neutralized XBB.1.5 along with its descendants EG.5.1 and HK.3. Neutralization titers against JN.1, however, are considerably lower. Due to the variant’s significant immune evasion and unprecedented transmissibility, both the World Health Organization (WHO) and the U.S. Food and Drug Administration (FDA) recommend vaccines against COVID-19 be formulated against the JN.1 antigen going forward. This guidance is reflective of the considerable antigenic differences between JN.1 and JN.1-lineage variants from their predecessors [8].

Animal models are a crucial component of disease research, enabling thorough investigation into viral pathogenesis and host–pathogen interactions. They enable the replication of clinical disease and associated pathology and are central to the preclinical evaluation of therapeutics, vaccines, monoclonal antibodies, and other countermeasures [8,9]. Mice are the most common animal model for biomedical research owing to their cost-effectiveness, ease of care, short reproductive cycles, ease of genetic manipulation, and genetic and biologic similarities to humans. Wild-type mice are not naturally permissive to infection with SARS-CoV or the ancestral strain of SARS-CoV-2 but can be rendered susceptible through the transgenic expression of human ACE2 (hACE2), the receptor required for host cell entry. Initially developed to model lethal SARS-CoV infection, the K18-hACE2 mouse model expresses hACE2 under the control of the epithelial cell cytokeratin-18 (K18) promoter, allowing for high-level expression of hACE2 in epithelial cells [10]. These mice are highly susceptible to SARS-CoV-2 infection, characterized by weight loss, severe respiratory disease, neurological manifestations, and mortality. We have previously established SARS-CoV-2 infection is lethal in the K18-hACE2 model and is accompanied by lung pathology, elevated proinflammatory cytokine production, and detectable viral RNA expression in the brain, lung, olfactory bulb, and nasal turbinates [9,11]. The K18-hACE2 model has been widely utilized to characterize differential pathogenesis of a significant number of SARS-CoV-2 variants, offering valuable insights into variant-specific disease outcomes and supporting preclinical evaluation of vaccines and therapeutics. The pathogenesis of viruses belonging to the JN.1 lineage, however, has not yet been fully investigated in this model. This represents a significant knowledge gap, as JN.1, the progenitor of all currently circulating variants, is antigenically distinct from earlier SARS-CoV-2 strains like XBB.1.5. The emergence of JN.1 marked a dramatic shift in SARS-CoV-2 evolution, and in vivo characterization of JN.1-lineage variants is essential for understanding how such antigenic divergence may alter disease outcomes and clinical manifestations, host immune responses, infection-induced pathology, and efficacy of vaccines, therapeutics, and other countermeasures.

In this study, we evaluated the pathogenicity of the SARS-CoV-2 Omicron subvariant JN.1 and its descendant KP.2 in comparison to the previously dominant XBB-lineage subvariant EG.5.1. Using the K18-hACE2 mouse model, we investigated viral replication, tissue tropism, clinical disease outcomes, immune responses, and histopathological changes to define variant-specific pathogenesis.

## 2. Materials and Methods

### 2.1. Cells and Viruses

Vero E6-TMPRSS2-T2A-ACE2 cells were obtained from BEI resources (NR-54970) and cultured in Dulbecco’s Modified Eagle’s medium (DMEM) supplemented with 10% fetal bovine serum (FBS) and 2% penicillin-streptomycin at 37 °C and 5% CO_2_.

The SARS-CoV-2 Omicron subvariants EG.5.1 (BEI Resources, NIAID, NIH: SARS-Related Coronavirus 2, Isolate hCoV-19/USA/MD-HP47946/2023 (Lineage EG.5.1; Omicron Variant), NR-59503), KP.2 (BEI Resources, NIAID, NIH: SARS-Related Coronavirus 2, Isolate hCoV-19/USA/CA-GBW-GKISBBBB26982/2024 (Lineage KP.2), NR-59890), and JN.1 (BEI Resources, NIAID, NIH: SARS-Related Coronavirus 2, Isolate hCoV-19/USA/New York/PV96109/2023 (Lineage JN.1; Omicron Variant), NR-59693) were propagated in Vero E6-TMPRSS2-T2A-ACE2 cells. Briefly, 24 h prior to infection, T-25 culture flasks were seeded with 7 × 10^5^ cells in 5 mL DMEM supplemented with 2% FBS and 2% penicillin-streptomycin and incubated at 37 °C and 5% CO_2_. Flasks were visually confirmed to be ~60% confluent immediately prior to infection. Media was aspirated from the flasks and replaced with fresh DMEM without additives. Initial virus stocks obtained from BEI were thawed and vortexed prior to inoculation of flasks with 100 μL of virus. Flasks were then incubated for 48 h at 37 °C and 5% CO_2_. Virus-containing supernatant was then collected, aliquoted, and stored at −80 °C for later use for viral challenge experiments. No additional sequencing was performed on the propagated virus stocks. To determine viral titers, 6-well tissue culture plates were seeded with 2 × 10^6^ cells/well in 2 mL of media. 10-fold dilutions of virus stocks were prepared in DMEM, and 100 μL of each dilution was used to inoculate appropriate wells. Plates were incubated for 1 h, swirling every 15 min. Cells were overlayed with a 1:1 solution of media and 2% agarose solution in diH_2_O and incubated for 48 h. A second overlay containing 2% neutral red was applied to facilitate plaque visualization. After an additional 24 h incubation, plaques were visualized and enumerated to determine viral titters. All work involving live SARS-CoV-2 was performed in a certified Biosafety Level 3 (BSL-3) laboratory at Georgia State University.

### 2.2. Mice

All animal experiments involving SARS-CoV-2 were performed in a certified Animal Biosafety Level 3 (ABSL-3) laboratory at Georgia State University. Protocols were approved by the GSU Institutional Animal Care and Use Committee (Protocol #A24003). Hemizygous K18-hACE2 (2B6.Cg-Tg (K18-ACE2)2Prlmn/J) mice aged six weeks were inoculated intranasally with 10^6^ plaque-forming units (PFU) of SARS-CoV-2 as described previously using the Omicron subvariants EG.5.1 (BEI# NR-59503), JN.1 (BEI# NR-59693), and KP.2 (BEI# NR-59890). Approximately equal numbers of male and female mice were used in this study. Animals were weighed and monitored for clinical disease daily. Mice found to be moribund or exhibiting 20% or greater loss of body weight were considered to meet humane endpoint criteria and euthanized. In separate experiments, mice were intranasally inoculated with indicated SARS-CoV-2 variant strains or PBS (mock) and sacrificed at 3 and 6 days post infection. Animals were anesthetized using isoflurane and perfused with 1×PBS or 4% paraformaldehyde (PFA) via cardiac puncture. Brain, lung, and nasal turbinate tissues were collected for further analysis.

### 2.3. RNA Extraction and Quantitative RT-PCR

Total RNA was extracted from harvested tissues using the Qiagen RNeasy Mini Kit (Qiagen, Venlo, The Netherlands, Cat# 74104) according to the manufacturer’s instructions and quantified using a Nanodrop microvolume spectrophotometer (ThermoFisher, Norcross, GA, USA). cDNA was synthesized from 1000 ng/μL of RNA using the iScriptTM Advanced cDNA Synthesis Kit for RT-qPCR (Bio-Rad, Hercules, CA, USA). The resulting cDNA was diluted with RNAse-free water, and 2 μL of cDNA was used per RT-qPCR. Viral RNA levels were quantified using primers and probes specific for the SARS-CoV-2 nucleocapsid (N) gene (Qiagen Cat# 222015) [10]. Viral genome copies were quantified by comparison to a standard curve generated using a known amount of RNA extracted from previously titrated SARS-CoV-2 samples [9].

### 2.4. Infectious Virus Titration via Plaque Assay

Harvested tissues were weighed and homogenized via a bead mill, followed by centrifugation and titration. Viral titers in tissue homogenates were measured via plaque assay performed on Vero E6-TMPRSS2-T2A cells. Cells were seeded in 6-well plates (5.0 × 10^5^ cells/well) and incubated for 24 h at 37 °C to form a monolayer. Tissue homogenates were serially diluted 10-fold in DMEM and applied to wells. Plates were incubated for 1 h at 37 °C and overlaid with 2% agarose. After 48 h of incubation, a second overlay containing 2% neutral red was applied to facilitate plaque visualization [9,12].

### 2.5. Histopathology

Harvested tissues were fixed in 4% PFA at room temperature for 24–48 h, washed with 1×PBS, and transferred to a 30% sucrose in 1×PBS for cryoprotection. Tissues were embedded in optimal cutting temperature (OCT) compound (Tissue-Tek, Torrance, CA, USA), frozen, and subsequently cut into 5–10 μm sections using a cryostat and affixed to charged slides for hematoxylin and eosin staining performed according to manufacturer instructions (Abcam, Cambridge, UK, Cat# ab245880) [9,13]. Images were captured using the Invitrogen EVOSTM M5000 Cell Imaging System (Invitrogen, Carlsbad, CA, USA).

### 2.6. Immunofluorescence Staining

Before staining, frozen slides were warmed at room temperature for 30 min and fixed in cold acetone for 10 min and allowed to air dry. Sections were thoroughly rinsed with PBS-T (0.1% Tween-20 in 1×PBS) and incubated with blocking buffer (PBS-T containing 5% goat serum) for 1 h. Slides were again washed with two changes in PBS-T prior to incubation with primary rabbit anti-SARS-CoV-2 nucleocapsid antibody (Cell Signaling Technologies, 1:500 dilution in blocking buffer) overnight at 4 °C. Following primary antibody incubation, slides were washed with PBS-T and incubated with Alexa Flour 555-conjugated secondary goat anti-rabbit antibody (Invitrogen, 1:500 dilution in blocking buffer) for 30 min. Slides were rinsed with PBS-T, incubated with DAPI for 5 min, rinsed, and mounted using ProlongTM Glass Antifade Mountant [12,14]. Images were captured using the Invitrogen EVOSTM M5000 Cell Imaging System.

### 2.7. Flow Cytometry Analysis

Mice were euthanized at 3 and 6 days post-infection (dpi) with isoflurane and perfused with 1×PBS via cardiac puncture. Lung single-cell suspensions were generated using the gentle MACS tissue dissociator (Miltenyi Biotec, Gladbach, Germany, Cat# 130-093-235). Single-cell suspensions were incubated with Fc Block antibody (BD Pharmingen, San Jose, CA, USA) in BD FACSTM Pre-Sort Buffer (BD Biosciences Cat# 563503) for 10 min. Cells were then incubated with antibodies against the following markers: PerCP Rat Anti-Mouse CD45 (BD Biosciences Cat# 557235), Pe-Cy5.5 Rat Anti-Mouse CD4 (BD Biosciences Cat# 550954), PE-Texas Red Rat Anti-Mouse CD8β (BD Biosciences Cat# 550798), APC-Cy™7 Rat Anti-Mouse CD11b (BD Biosciences Cat# 561039), PE-Texas Red CD11c (Thermofisher Scientific, Cat# MCD11C17), and BD HorizonTM Fixable Viability Stain 575V (BD Biosciences Cat# 565694). Cells were stained for 30 min on ice and fixed (eBioscience, San Diego, CA, USA) according to the manufacturer instructions. Flow cytometry data was obtained using the BD LSRFortessa™ Cell Analyzer, and subsequent data analysis was performed using FlowJo software (version 11), as previously described [15].

### 2.8. Statistical Analysis

Statistical analysis was performed using GraphPad Prism version 10.0. Survival curves were generated using the Kaplan–Meier method, and statistical comparisons between variant groups were performed using the log-rank (Mantel–Cox) test. Differences in body weight loss data were analyzed using a mixed-effects model followed by Tukey’s multiple comparison test. Statistical significance for viral titers and RT-qPCR data was determined by either using one-way ANOVA with Tukey’s post hoc analysis or the Kruskal–Wallis test with Dunn’s multiple comparisons test. Differences of *p* < 0.05 were considered statistically significant.

## 3. Results

### 3.1. Clinical Disease Outcomes Following Intranasal Infection of K18-hACE2 Mice with SARS-CoV-2 Subvariants JN.1, KP.2, and EG.5.1

**Figure 1:**
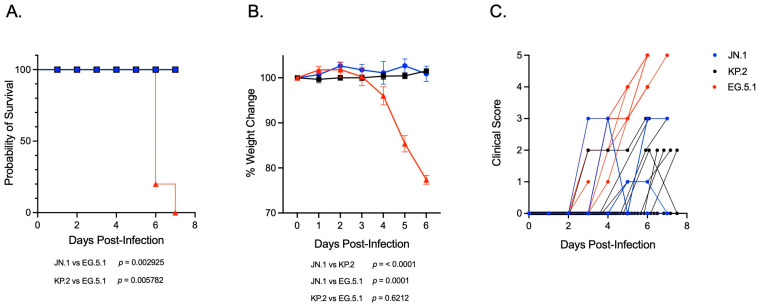
Disease severity and clinical outcomes in K18-hACE2 mice infected with SARS-CoV-2 variants JN.1, KP.2, and EG.5.1. Six-week-old K18-hACE2 mice were intranasally inoculated with 10^6^ plaque-forming units (PFU) of either JN.1, KP.2, or EG.5.1. (**A**) Kaplan–Meier survival curve showing percent survival mice infected with JN.1 (*n* = 17), KP.2 (*n* = 15), and EG.5.1 (*n* = 14). Statistical significance is determined using the log-rank (Mantel–Cox) test (*p* = 0.0006). Pairwise comparisons demonstrated significantly reduced survival in EG.5.1-infected mice compared to both JN.1 (*p* = 0.0029) and KP.2 (*p* = 0.0057). (**B**) Body weight was measured daily following infection. Weights are expressed as the percentage of initial body weight (Day 0). Data are represented as means ± SEM. Statistical significance was assessed using a mixed-effects model with Tukey’s multiple comparisons test. Significant differences were observed between EG.5.1 and both JN.1 (*p* < 0.0001) and KP.2 (*p* = 0.001). No significant difference was found between JN.1 and KP.2. (**C**) Clinical scores between 0 and 5 assigned based on observed symptoms (0 = no symptoms, 1 = lethargic, slow movement, 2 = ruffled fur, 3 = hunched posture, 4 = rounded posture, shaking, 5 = moribund, labored breathing, paralysis/walking disability).

To investigate and compare the pathogenicity of SARS-CoV-2 Omicron subvariants belonging to JN.1- and XBB-lineages in vivo, six-week-old K18-hACE2 mice were inoculated intranasally with 10^6^ PFU (plaque-forming units) of either JN.1, the JN.1-derived subvariant KP.2, or the XBB-lineage subvariant EG.5.1. Mice were weighed daily and monitored for survival and clinical signs of disease. Infection with JN.1 or KP.2 was non-lethal in K18-hACE2 mice, with an observed survival rate of 100% in both groups (Figure 1A). Significant weight loss was not observed following infection with either JN.1-lineage subvariant (Figure 1B). While most animals remained asymptomatic, a small subset of mice in both groups developed mild clinical symptoms, such as ruffled fur and reduced activity 2–3 days post inoculation (dpi), which typically resolved within two days of onset (Figure 1C). In contrast, infection with the EG.5.1 resulted in 100% mortality in K18-hACE2 mice within 7 days, accompanied by a marked increase in disease severity (Figure 1A,B). By 4 dpi, EG.5.1-infected mice displayed weight loss and moderate clinical symptoms (e.g., ruffled fur, hunched posture, and reduced activity). Rapid disease progression was evident by 5 dpi, characterized by a dramatic increase in symptom severity (e.g., rounded posture, shaking, limb paralysis, respiratory distress) and further weight loss. All mice infected with EG.5.1 succumbed to infection or reached humane endpoint criteria for euthanasia (e.g., >20% weight loss, moribund) within 6–7 dpi (Figure 1B,C). Mixed-effects analysis confirmed weight loss to be significantly greater following infection with EG.5.1 compared to both JN.1-lineage variants. Log-rank analysis demonstrated significantly increased mortality in EG.5.1-infected mice compared to both JN.1 (*p* = 0.0029) and KP.2 (*p* = 0.0057), corresponding with the observed differences in body weight loss and clinical disease severity. The observation of lineage-specific disease phenotypes suggests attenuated pathogenicity of JN.1-lineage variants in vivo compared to the XBB-derived EG.5.1.

### 3.2. Assessment of Viral Burden in JN.1-, KP.2-, and EG.5.1-Infected Tissues

**Figure 2:**
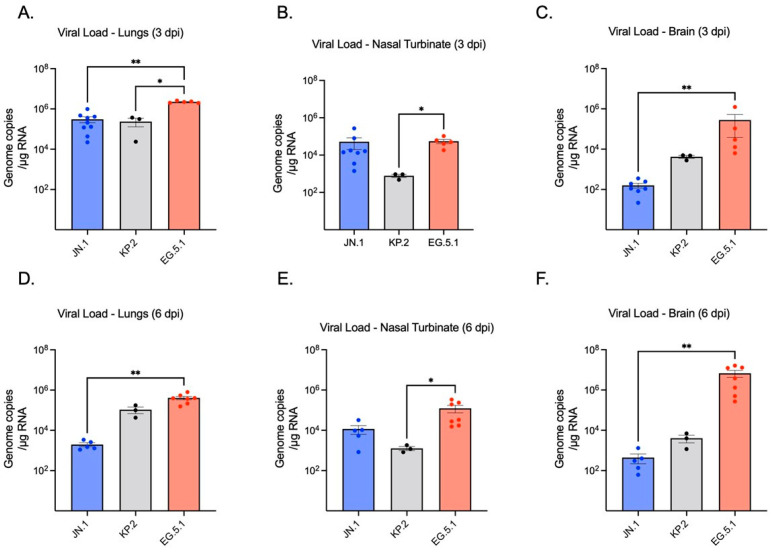
Quantification of SARS-CoV-2 viral RNA detected in lung, brain, and nasal turbinates of K18-hACE2 mice at 3 and 6 days post-infection (dpi). Six-week-old K18-hACE2 mice were intranasally inoculated with 10^6^ plaque-forming units (PFU) of either JN.1, KP.2, or EG.5.1 in independent experiments. Groups of mice (*n* = 3–9) were euthanized at 3 dpi and 6 dpi, tissues were collected, and viral RNA levels were quantified by RT-qPCR. (**A**,**D**) Viral RNA in infected lung tissues (**B**,**E**) Viral RNA in nasal turbinate tissues (**C**,**F**) Viral RNA present in brain turbinate tissues. Viral load is expressed as genomic copies/μg of total RNA. Each point represents an individual mouse. Data are presented as mean ± SEM for each variant group. Statistical significance was assessed using either a Kruskal–Wallis test with Dunn’s multiple comparisons or a one-way ANOVA with Tukey’s post hoc test (* *p* < 0.05; ** *p* < 0.01).

**Figure 3:**
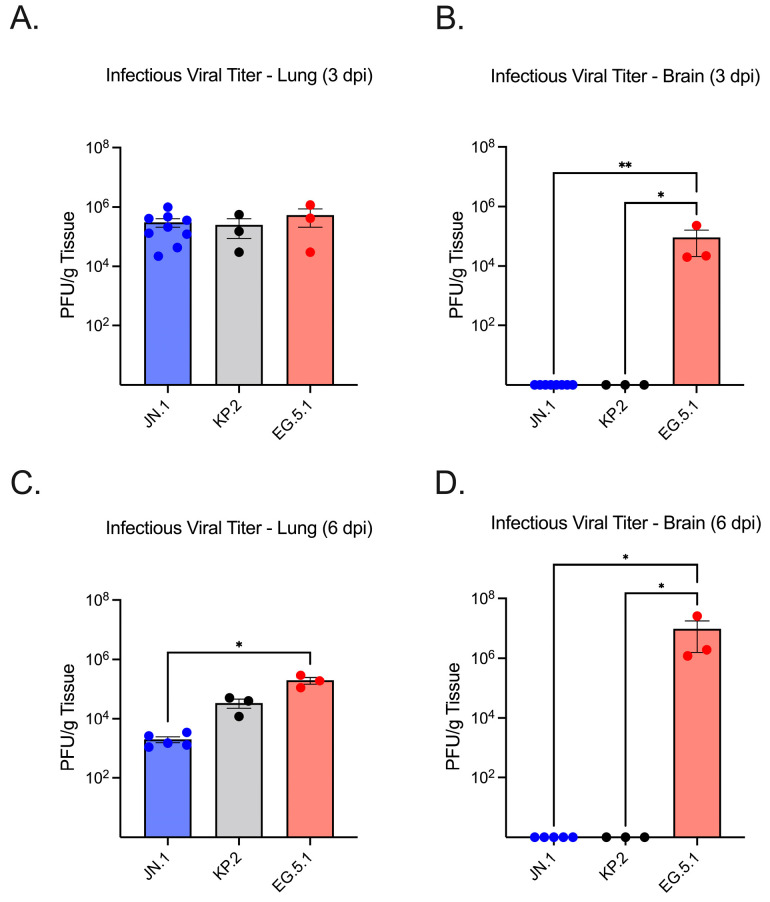
Infectious viral titers in lung and brain tissues of K18-hACE2 mice infected with SARS-CoV-2 variants. Six-week-old mice were intranasally inoculated with 10^6^ PFU of either JN.1, KP.2, or EG.5.1 and euthanized at 3 and 6 days post-infection (*n* = 3–9). Lung and brain tissues were collected, homogenized, and used to quantify infectious virus via plaque assay, expressed as PFU/gram of tissue. (**A**) Infectious virus titers in lung tissues collected at 3 dpi. (**B**) Infectious virus titers in brain tissues collected at 3 dpi. (**C**) Infectious virus titers in lung tissues collected at 6 dpi. (**D**) Infectious virus titers in brain tissues collected at 6 dpi. Each data point represents an individual mouse. Bars indicate the mean ± SEM for each variant group. Statistical significance was determined using one-way ANOVA with Tukey’s post hoc analysis or the Kruskal–Wallis test followed by Dunn’s multiple comparisons test (* *p* < 0.05; ** *p* < 0.01).

To assess viral replication in the lower respiratory tract, SARS-CoV-2 RNA levels in lung tissue homogenates were quantified by RT-qPCR. No significant difference was observed between JN.1- (2.10 × 10^5^ genome copies/g RNA ± SEM) and KP.2-infected lung tissues (3.13 × 10^5^) at 3 dpi, whereas EG.5.1-infected lungs (2.21 × 10^6^) exhibited significantly higher viral RNA levels compared to both JN.1 (*p* ≤ 0.0001) and KP.2 (*p* ≤ 0.0001) (Figure 2A). By 6 dpi, a marked decrease in viral RNA was seen across variants. Highest levels of viral RNA were consistently observed in EG.5.1-infected tissues (3.92 × 10^5^). A significant difference was observed between EG.5.1-infected tissues and KP.2 tissues (*p* = 0.0344), while RNA levels in EG.5.1-infected tissues were found to be approximately 100-fold higher than in JN.1-infected tissues (1.50 × 10^3^) (*p* = 0.0018) (Figure 2D). These findings were consistent with infectious virus titers measured by plaque assay (Figure 3A,C). Viral titers were highest at 3 dpi for all variants, with no significant differences between EG.5.1- (4.20 × 10^5^ PFU/g ± SEM), KP.2- (1.50 × 10^5^), and JN.1-infected tissues (2.10 × 10^5^). A considerable decline in viral titers was seen by 6 dpi, with the lowest levels in JN.1-infected lung tissue (1.50 × 10^3^ PFU/g tissue ± SEM) and the highest in EG.5.1-infected tissues (1.90 × 10^5^) (*p* = 0.0011). A significant difference was also observed between KP.2- and EG.5.1-infected tissues (*p* = 0.0064). Together, these results indicate more efficient viral replication by EG.5.1 than both JN.1-lineage variants in the lower respiratory tract of K18-hACE2 mice.

Viral replication in the upper respiratory tract was measured using nasal turbinate tissues of infected mice collected at 3 and 6 dpi, finding a significant difference observed between variants. At 3 dpi, the highest viral RNA levels were measured in EG.5.1-infected tissues (5.02 × 10^4^). KP.2-infected tissues (9.25 × 10^2^) exhibited the lowest levels of viral RNA, with a 54-fold decrease compared to EG.5.1 (*p* = 0.0101) and a 16-fold decrease when compared to JN.1 (1.52 × 10^4^) (Figure 2B,E). On day 6, slightly lower levels of viral RNA were detected in EG.5.1- (3.31 × 10^4^) and JN.1-infected tissues (9.69 × 10^3^), and KP.2-infected tissues (1.18 × 10^3^), showing significantly lower viral RNA levels compared to EG.5.1-infected tissues (*p* = 0.0117).

We next evaluated neuroinvasion by JN.1, KP.2, and EG.5.1. Consistent with other XBB-lineage variants, viral RNA was readily observed in EG.5.1-infected brain tissues as soon as 3 dpi (2.95 × 10^2^), followed by a substantial increase in viral load by 6 dpi (3.80 × 10^6^) (Figure 2C,F). Viral RNA was significantly higher in EG.5.1-infected mice compared to JN.1-infected mice at both timepoints (*p* = 0.0018 and *p* = 0.0023, respectively). These results were supported by plaque assay, which revealed infectious titers of 2.20 × 10^4^ at day 3 and 1.9 × 10^6^ at day 6 (Figure 3C,F). Low levels of genomic viral RNA were detected in the brain tissues of JN.1- and KP.2-infected mice at both timepoints; however, no infectious virus was detected by plaque assay. This indicates that unlike prior SARS-CoV-2 variants and the XBB-lineage subvariant EG.5.1, JN.1-derived variants are not neuroinvasive in the K18-hACE2 mouse model.

### 3.3. Histological Analysis and Viral Antigen Expression

**Figure 4:**
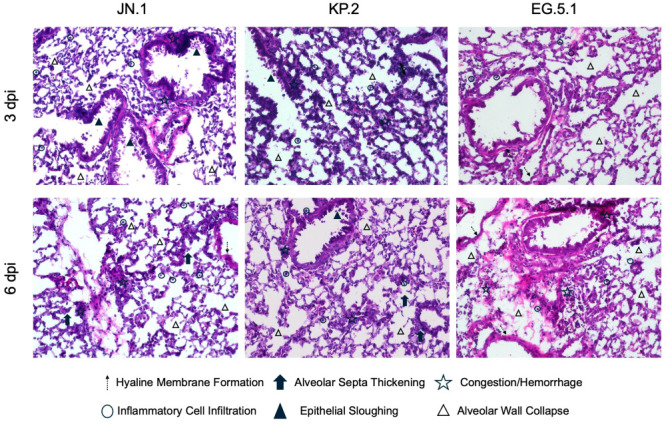
Histopathological changes following SARS-CoV-2 variant infection. Six-week-old K18-hACE2 mice were intranasally inoculated with 10^6^ plaque-forming units (PFU) of either JN.1, KP.2, or EG.5.1 in independent experiments. Lung tissues were collected at 3 and 6 days post infection for immunofluorescence staining and histopathological analysis. Representative images of hematoxylin and eosin (H&E)-stained lung tissues at 20X magnification. Images illustrate pathological changes, including altered alveolar structure, inflammatory cell infiltration, congestion, epithelial sloughing, hemorrhage, and hyaline membrane formation within infected tissues compared to mock-infected control tissues.

In independent experiments, six-week-old K18-hACE2 mice were intranasally inoculated with JN.1, KP.2, or EG.5.1 and euthanized at 3 dpi and 6 dpi. Lung and brain tissues were collected for histological analysis via hematoxylin and eosin (H&E) staining (Figure 4). For all variants, lung tissues collected from JN.1-, KP.2-, and EG.5.1-infected mice displayed significant pathological changes at both timepoints. At 3 dpi, moderate immune cell infiltration, peribronchial cuffing, and vascular congestion were observed across variants. Pathology was most pronounced in EG.5.1-infected lung tissues, showing marked alterations to alveolar structure compared to JN.1-lineage-infected tissues. At 6 dpi, severe pathological changes were observed in JN.1-, KP.2-, and EG.5.1-infected tissues, including prominent immune cell infiltration, perivascular and peribronchial cuffing, consolidation, and vascular congestion. Pathology was most pronounced in EG.5.1-infected tissues with extensive changes to alveolar structure, vascular congestion, consolidation, and evidence of alveolar hemorrhage.

**Figure 5:**
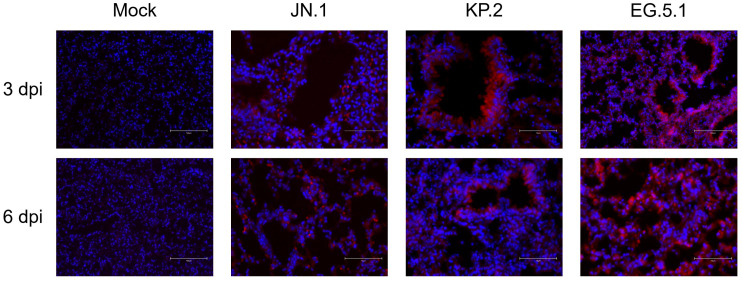
Viral antigen expression following SARS-CoV-2 variant infection. Six-week-old K18-hACE2 mice were intranasally inoculated with PBS (mock) or with 10^6^ plaque-forming units (PFU) of either JN.1, KP.2, or EG.5.1 in independent experiments, and lung tissues were collected at 3 and 6 days post infection. Lung tissues were stained with primary rabbit anti-mouse SARS-CoV-2 nucleocapsid antibody, incubated with Alexa Fluor 555-conjugated goat anti-rabbit secondary antibody (red), and counterstained with DAPI (blue). Scale bars: 75 μm.

Immunofluorescence (IF) staining was used to visualize viral antigen distribution in brain and lung tissues at 3 and 6 dpi. For all variants, SARS-CoV-2 nucleocapsid protein and DAPI were readily detected in lung tissues at both timepoints. Nucleocapsid signal was most concentrated around the bronchioles, suggesting high amounts of viral antigen are present in this region throughout infection (Figure 5).

### 3.4. Cellular Response in the Lungs Following EG.5.1 Infection

**Figure 6:**
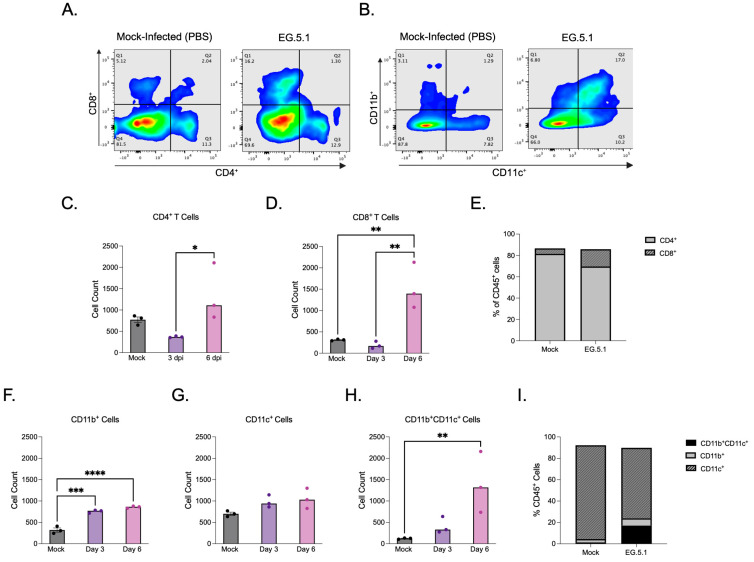
Cellular immune response to EG.5.1 infection. Flow cytometric analysis of lung tissues in K18-hACE2 mice following intranasal inoculation with 10^6^ PFU of EG.5.1 or mock infection (PBS) at 3 and 6 dpi. (**A**) Representative expression of CD4^+^ T cells and CD8^+^ T cells visualized by surface staining on CD45^+^ cells in mock- and EG.5.1 -infected lungs. (**B**) Representative expression of CD11b^+^ cells, CD11c^+^ cells and CD11b^+^CD11c^+^ cells visualized by surface staining on CD45^+^ in mock- and EG.5.1-infected lungs. (**C**) CD4^+^ T cell numbers. (**D**) CD8^+^ T cell numbers. (**E**) Frequency of CD4^+^ and CD8^+^ T cells within the CD45^+^ populations of lungs of mock- and EG.5.1 infected mice at 6 dpi. (**F**) CD11b^+^ myeloid cell numbers. (**G**) CD11c^+^ myeloid cell numbers (**H**) CD11c^+^CD11b^+^ myeloid cell numbers. (**I**) Frequency of CD11b^+^, CD11c^+^, and CD11b^+^CD11c^+^ myeloid subsets within the CD45^+^ population in the lungs of mock- and EG.5.1-infected mice at 6 dpi. Each data point represents an individual mouse. Bars indicate the mean ± SEM. *n* = 3 for both mock-infected and EG.5.1-infected animals at 3 and 6 dpi. Significance was determined by using a one-way ANOVA followed by Tukey’s multiple comparisons test or by using the Kruskal–Wallis test followed by Dunn’s multiple comparisons test (* *p* < 0.05; ** *p* < 0.01; *** *p* < 0.001, **** *p* < 0.0001).

The XBB-lineage variant EG.5.1 was selected for in-depth immune cell analysis due to its association with the most severe disease phenotype among variants tested, characterized by significant weight loss and mortality accompanied by extensive lung histopathology, high levels of viral antigen expression, infectious virus, and genomic RNA detected within infected tissues. Additionally, EG.5.1 has not yet been fully characterized in the K18-hACE2 mouse model, further motivating interest in examining the immune response elicited by infection. Flow cytometry was performed on lung tissues collected from K18-hACE2 mice infected with EG.5.1 at 3 and 6 days post-infection. CD45^+^ cells were used to gate immune cell populations, and both lymphoid and myeloid subsets were quantified (Figure 6A,B). Flow cytometric analysis revealed dynamic changes in T cell populations during infection. At 3 dpi, lower numbers of CD4^+^ and CD8^+^ T cells were observed in EG.5.1-infected lungs compared to mock-infected control lungs, potentially suggesting a weakened or delayed early immune response (Figure 6C,D). At 6 dpi, a significant increase in both CD4^+^ and CD8^+^ T cells was observed in EG.5.1-infected tissues compared to 3 dpi (*p* = 0.0113 and *p* = 0.0048, respectively) (Figure 6C–E).

To further characterize the pulmonary immune response to EG.5.1 infection, myeloid cell populations within lung tissues were analyzed based on CD11b and CD11c expression profiles. We detected a significant increase in the number of CD11b^+^CD11c^−^ cells in EG.5.1-infected lungs at day 3 and 6 days post infection compared to mock-infected controls (Figure 6F). We also detected a slight increase in the number of CD11c^+^CD11b^−^ cells in EG.5.1-infected lungs at day 3 and 6 days post infection compared to mock-infected controls; however, the difference was not statistically significant (Figure 6G). The number of double positive CD11b^+^CD11c^+^ cells increased significantly by day 6 post infection (Figure 6H,I). These results suggest the active recruitment and/or differentiation of inflammatory dendritic cells in response to viral infection.

## 4. Discussion

The rapid ongoing evolution of SARS-CoV-2 remains a challenge to public health, particularly as new variants alter viral fitness, replication dynamics, tissue tropism, and clinical disease outcomes. As therapeutic, preventative, and diagnostic strategies are re-evaluated, it is equally important to assess how emerging variants interact within established animal models [10,12,13]. Animal models are crucial tools for preclinical research and are essential for understanding variant-specific pathogenesis, testing countermeasures, and predicting disease outcomes. In this study, we characterized the pathogenesis of SARS-CoV-2 Omicron subvariants JN.1, its direct descendant KP.2, and the XBB-lineage variant EG.5.1 in the K18-hACE2 mouse model. These variants were selected based on their evolutionary importance, with JN.1 representing the current parental lineage of all currently circulating strains globally and EG.5.1 representing the previously dominant XBB lineage [2,14]. Our results demonstrate remarkable lineage-specific differences in clinical disease, viral replication, neuroinvasion, and immune response, underscoring the importance of continued variant-specific pathogenesis studies. Such research is critical not only for understanding the biology of the recently evolved JN.1 lineage but also for informing vaccine reformulations, therapeutic preparedness, interpretation of variant-driven clinical trends, and providing insight into the ongoing evolution of SARS-CoV-2.

We have previously demonstrated that infection with the parental Omicron variant (B.1.1.529) results in attenuated disease in K18-hACE2 mice, whereas the subsequently dominant Omicron subvariant XBB.1.5 induces severe pulmonary pathology and high mortality [9,11,15]. In this study, a clear divergence in pathogenesis was observed between the JN.1-lineage variants JN.1 and KP.2 and the XBB-lineage variant EG.5.1. Infection with JN.1 or KP.2 was non-lethal in K18-hACE2 mice, characterized by mild, transient clinical disease symptoms and the absence of significant weight loss. These findings are consistent with the decreased disease severity observed in humans infected with JN.1-lineage variants [6,16,17]. Similarly, independent studies have shown that JN.1 causes only mild disease, with no significant weight loss or mortality observed in mice and hamsters. Additional studies have demonstrated that BA.2-descendant variants, including BA.2.86 and JN.1, replicate less efficiently and induce less lung injury than XBB-descendant variants such as EG.5.1 and HK.3 [17,18,19]. Although the JN.1 mutation L455S enhances transmissibility and immune evasion, it simultaneously reduces ACE2 binding affinity, which may contribute to the attenuated disease observed with the JN.1 lineage [20,21].

Despite the non-lethal outcome, robust viral replication was observed in both upper and lower respiratory tract tissues following infection. Viral replication was consistently higher in KP.2-infected tissues compared to JN.1-infected tissues at both timepoints, which is consistent with previous reports demonstrating the increased fitness and replication efficiency of KP.2 compared to JN.1 [4,22,23,24]. By contrast, infection with EG.5.1 resulted in the rapid development of severe clinical disease, ultimately resulting in 100% mortality, closely resembling the lethal disease phenotype we have previously reported with XBB.1.5 [9]. Viral RNA levels detected in the lungs and nasal turbinates of EG.5.1-infected mice were consistently higher than those infected with JN.1-lineage subvariants. These findings were further supported by the viral plaque assay, with the highest levels of infectious virus found within EG.5.1-infected tissues, indicating more effective replication within the respiratory tissues compared to JN.1-lineage variants.

Although most SARS-CoV-2 infections are mild, severe disease can result from dysregulated host immune responses characterized by uncontrolled viral replication and excessive inflammation, with some cases progressing to acute respiratory distress syndrome (ARDS), which may lead to death [8,19,25]. Consistent with severe pathological changes observed in human cases of COVID-19, H&E staining of EG.5.1-infected lungs revealed extensive pathology marked by diffuse alveolar damage, perivascular cuffing, congestion, thickening of alveolar septum, and immune cell infiltration. In contrast, lung tissues collected from JN.1- and KP.2-infected mice exhibited milder pathology at both timepoints compared to EG.5.1-infected mice, correlating with lower viral loads. Together, these findings suggest EG.5.1 induces more severe and widespread lung pathology than JN.1-lineage variants in this model, consistent with clinical observations in humans [26].

While SARS-CoV-2 infection is primarily associated with respiratory disease, neurobiological involvement is well documented in both humans and animal models [27,28,29]. Human autopsy studies have identified the presence of SARS-CoV-2 RNA transcripts in the brain, cerebrospinal fluid, and endothelial cells located within the olfactory bulb of deceased individuals [30]. Clinically, neurological manifestations range in severity from mild symptoms, including headache, anosmia, ageusia, dizziness, and cognitive impairment, to more severe outcomes, including seizure, stroke, encephalitis, encephalopathy, and peripheral neuropathy. Our findings indicate XBB- and JN.1-lineage variants differ in their tissue tropism and neuroinvasive potential. In EG.5.1-infected mice, both viral RNA and infectious virus were detected in brain tissues at both 3 and 6 dpi, consistent with our previous observations of neuroinvasion by XBB.1.5 in the K18-hACE2 model. These results indicate that, like other XBB-lineage variants, EG.5.1 is capable of neuroinvasion and productive infection of the central nervous system [9]. Alternatively, no infectious virus was detected in the brain tissues of mice infected with JN.1-lineage variants at either timepoint despite robust replication in the upper and lower respiratory tracts. This suggests JN.1-lineage variants exhibit altered tissue tropism relative to earlier Omicron variants and former SARS-CoV-2 variants of concern. Together, these data highlight the neurotropic potential of EG.5.1 and its capacity for extrapulmonary infection. The absence of neuroinvasion by JN.1-lineage viruses in this model further supports their attenuated phenotype.

The host immune response is a critical determinant of COVID-19 disease severity and outcome. Notably, T cell lymphopenia has been associated with more severe clinical presentation, emphasizing the essential role of cellular immunity in the control and clearance of SARS-CoV-2 infection [31,32]. In K18-hACE2 mice, EG.5.1 infection led to a marked reduction in pulmonary CD4^+^ and CD8^+^ T cells at 3 dpi when compared to mock-infected controls. This depletion is consistent with early T cell lymphopenia, suggesting a delayed or suppressed adaptive immune response during the initial stages of infection. By 6 dpi, a significant rebound was observed, particularly within the CD8^+^ T cell compartment, which surpassed levels seen in control tissues. These findings indicate a biphasic T cell response to EG.5.1 infection featuring an initial suppression and subsequent expansion. In contrast to the fluctuating T cell response, sustained myeloid cell infiltration was evident throughout infection. At 6 dpi, EG.5.1-infected lungs exhibited increased numbers of CD11b^+^, CD11c^+^, and CD11b^+^CD11c^+^ cells, indicating ongoing recruitment or differentiation of myeloid cell populations within the lung tissue. Together these observations illustrate a time-dependent shift in the pulmonary immune response to EG.5.1 infection.

While the K18-hACE2 mouse model provides valuable insight into SARS-CoV-2 pathogenesis, several limitations should be considered. In this model, human ACE2 (hACE2) expression is driven by the non-native cytokeratin-18 promoter, resulting in altered tissue expression compared to endogenous ACE2 expression in humans. Additionally, this model does not account for prior SARS-CoV-2 infection or vaccine-induced immunity, factors that significantly influence infection dynamics and disease outcomes in the human population. Further investigation is needed to determine the role of specific spike mutations in JN.1-lineage variants and how these contribute to lineage-specific differences in replication efficiency, virulence, and tissue tropism observed in this model.

In conclusion, we characterized the pathogenesis of SARS-CoV-2 Omicron subvariants JN.1, KP.2, and EG.5.1 in K18-hACE-2 mice. Our findings demonstrate that infection with JN.1-lineage variants result in significantly attenuated, non-lethal disease. In contrast, EG.5.1 infection led to severe pulmonary disease and 100% mortality. EG.5.1-infected tissues showed the highest levels of RNA and infectious virus in both the upper and lower respiratory tracts, with JN.1-infected tissues showing the lowest levels. The XBB-lineage variant EG.5.1 was found to be neuroinvasive, with viral RNA and infectious virus detected in brain tissues as early as 3 days after infection. In contrast, evidence of infectious virus was not detected in the brain of JN.1- or KP.2-infected mice. This observed lack of neuroinvasion likely reflects altered tissue tropism driven by the extensive spike mutations unique to JN.1-lineage viruses and is consistent with the attenuated phenotype observed in both animal models and human infections.

## Data Availability

The original contributions presented in the study are included in the article; further inquiries can be directed to the corresponding author.
